# Research progress on application of single-cell TCR/BCR sequencing technology to the tumor immune microenvironment, autoimmune diseases, and infectious diseases

**DOI:** 10.3389/fimmu.2022.969808

**Published:** 2022-08-18

**Authors:** Jinhua He, Jian Shen, Wenfeng Luo, Zeping Han, Fangmei Xie, Ting Pang, Liyin Liao, Zhonghui Guo, Jianhao Li, Yuguang Li, Hanwei Chen

**Affiliations:** ^1^ Central Laboratory, Central Hospital of Panyu District, Guangzhou, China; ^2^ Institute of Cardiovascular Medicine, Central Hospital of Panyu District, Guangzhou, China; ^3^ Administrative Office, He Xian Memorial Hospital, Southern Medical University, Guangzhou, China; ^4^ Medical Imaging Institute of Panyu, Central Hospital of Panyu District, Guangzhou, China

**Keywords:** single-cell TCR/BCR sequencing, autoimmune diseases, infectious diseases, Chronic inflammatory diseases, Tumor immune microenvironment

## Abstract

Single-cell omics is the profiling of individual cells through sequencing and other technologies including high-throughput analysis for single-cell resolution, cell classification, and identification as well as time series analyses. Unlike multicellular studies, single-cell omics overcomes the problem of cellular heterogeneity. It provides new methods and perspectives for in-depth analyses of the behavior and mechanism of individual cells in the cell population and their relationship with the body, and plays an important role in basic research and precision medicine. Single-cell sequencing technologies mainly include single-cell transcriptome sequencing, single-cell assay for transposase accessible chromatin with high-throughput sequencing, single-cell immune profiling (single-cell T-cell receptor [TCR]/B-cell receptor [BCR] sequencing), and single-cell transcriptomics. Single-cell TCR/BCR sequencing can be used to obtain a large amount of single-cell gene expression and immunomics data at one time, and combined with transcriptome sequencing and TCR/BCR diversity data, can resolve immune cell heterogeneity. This paper summarizes the progress in applying single-cell TCR/BCR sequencing technology to the tumor immune microenvironment, autoimmune diseases, infectious diseases, immunotherapy, and chronic inflammatory diseases, and discusses its shortcomings and prospects for future application.

## Introduction

**Figure 1:**
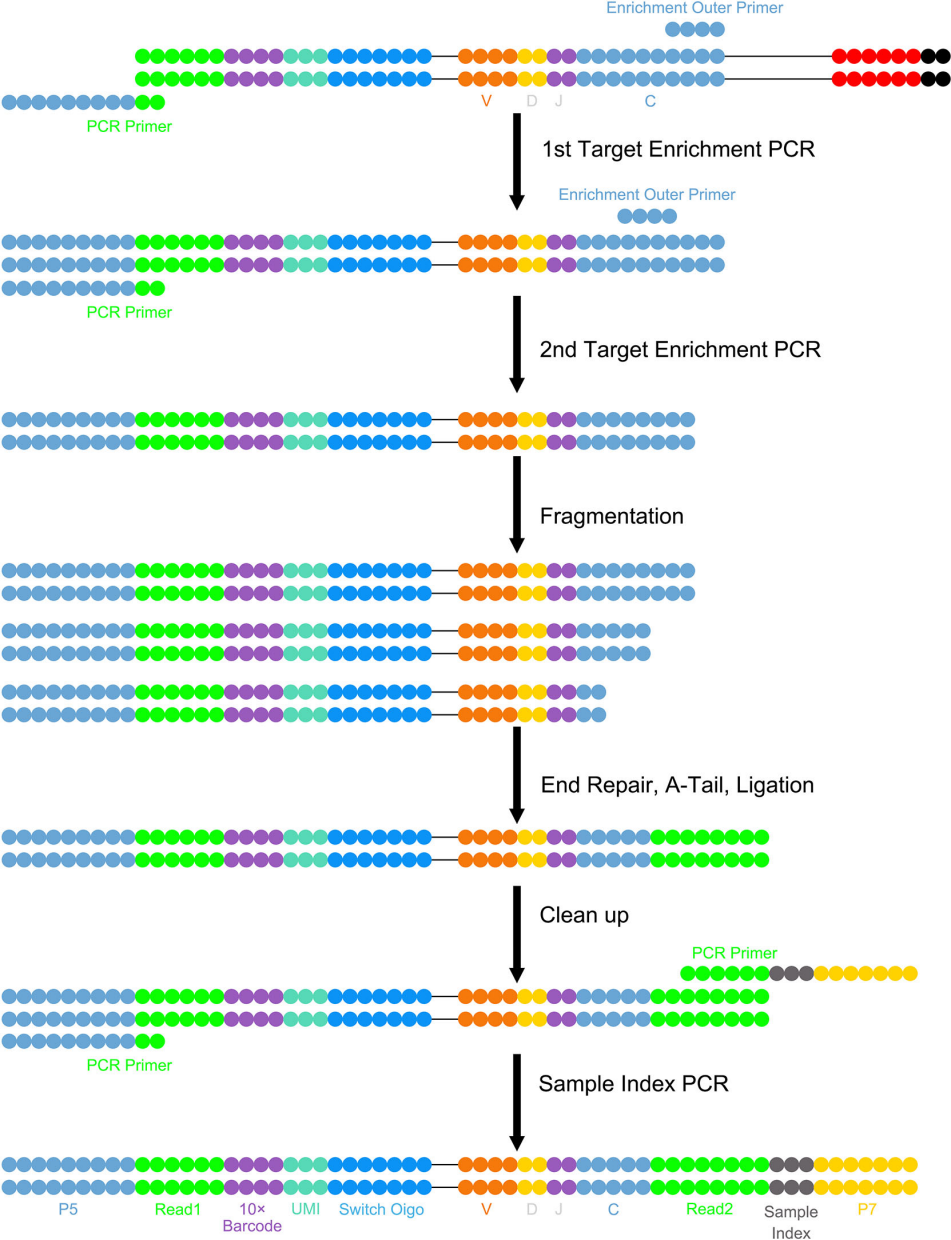
Schematic diagram of single-cell TCR/BCR sequencing technology.

The immune repertoire (IR) refers to the sum of B and T lymphocytes with functional diversity in an individual’s circulatory system at any point in time (1). T and B cells mediate the cellular and humoral immune responses of the body, and recognize and bind antigens through T-cell receptors (TCRs) and B-cell receptors (BCRs) on their respective surfaces to clear pathogens or tumor cells *in vivo* (2). IR sequencing (IR-seq) targets T and B lymphocytes. Multiplex PCR or 5’-rapid amplification of cDNA (complementary Deoxyribonucleic acid) ends was used to amplify the complementarity-determining region (CDR) that determines the diversity of TCR or BCR, combined with high-throughput sequencing technology, to comprehensively assess the diversity of the immune system and explore the relationship between the IR and disease (3). IR-seq technology mainly includes single-cell TCR and BCR sequencing; a schematic illustration is shown in **Figure 1**.

Single-cell TCR sequencing by high-throughput sequencing technologies allows detection of the target after amplification and recognition of surface T-cell antigen, analysis of its diversity, and before and after T-cell antigen recognition, can reflect the body’s physiological and pathological conditions by detecting changes in T-cell mediated immune responses. Single-cell TCR sequencing can be used to study the transcription and interrelationships of different T-cell clones, thus revealing deeper T-cell functional specificity (4). Single-cell TCR sequencing detects the heavy and light BCR chains after targeted amplification by high-throughput sequencing technology, and comprehensively analyzes the rearranged base sequences of the BCR gene and abundance of each sequence. It is used to study the transcription and interrelationship of different B-cell clones, suggesting a deeper level of B-cell functional specificity, and thus explaining humoral immune response tolerance and high-frequency mutations in B-cell response recognition antigen-related abnormalities (5).

Single-cell TCR/BCR sequencing technology has the technical advantages of high throughput, high resolution, and comprehensive information, and has been comprehensively applied research of the tumor immune microenvironment, autoimmune diseases, infectious diseases, immunotherapy, chronic inflammation, and other diseases.

## Tumor immune microenvironment

Normal karyotype acute myeloid leukemia (NK-AML) is a highly heterogeneous malignancy that exists in a complex immune microenvironment. Understanding tumor-infiltrating T cells is critical for advancing immunotherapy and improving outcomes in patients with this disease. In one study, single-cell sequencing was performed on bone marrow cells from 5 patients with NK-AML (M4/M5) and 1 normal donor. The results showed that mucosa-associated constant T cells (MAITs) were preferentially enriched and likely to be clonally amplified in the NK-AML patients, providing valuable insights into the immune microenvironment of NK-AML (6). In another study, single-cell sequencing was used to analyze 45,000 immune cells from 8 breast cancer patients, as well as matched normal breast tissue, blood, and lymph nodes. The results showed that there was a continuous activation model in T cells and a macrophage polarization model that did not conform to cancer. Understanding immune cell phenotypes in the tumor microenvironment is of great significance in revealing the mechanisms of cancer progression and immunotherapy response (7). In patients with early-stage breast cancer, the degree of tumor-infiltrating lymphocytes (TILs) was associated with the response to chemotherapy and overall survival. Eighteen patients with early-stage breast cancer were treated preoperatively with cryoablation, single-dose anti-CTLA-4(cytotoxic T lymphocyte-associated protein), or cryoablation plus ipilimumab. Single-cell sequencing results showed that in basal tumor tissue, T-cell density as measured by TCR sequencing was associated with TIL degree score as measured by hematoxylin and eosin (H&E) staining. This provides a new direction for further research using TCR sequencing as a biomarker of T-cell response to treatment and cry immunotherapy for early-stage breast cancer (8). Severe immune-related adverse events (irAEs) occur in up to 60% of melanoma patients treated with immune checkpoint inhibitors (ICIs). TCR sequencing has been used to examine the T-cell repertoire in peripheral blood samples from melanoma patients receiving antitumor therapy. The results showed that the abundance of CD4 memory T cells is associated with the development of severe irAEs; thus, the distribution of related circulating T-cell characteristics induced by ICIs is of great significance for improving clinical diagnosis and management (9). In one study, single-cell RNA combined with TCR sequencing was used to detect “tumor-matched” (TM) CD8+ T cells in the blood of patients with melanoma, using TCR as a molecular barcode. The results showed that TM cells showed higher activation compared to mismatched T cells in the blood and were less depleted than matched cells in the tumor, which has great potential for monitoring anti-tumor CD8+ T cell responses in the blood (10). The detection of TILs is the key to developing immunotherapy and predicting its clinical response in cancer. TCR sequencing analysis of 5,063 T cells isolated from the peripheral blood, tumors, and nearby normal tissues of 6 patients with hepatocellular carcinoma (HCC) showed that depleted CD8+ T cells and regulatory T cells (Tregs) were preferentially enriched and clonally amplified in HCC. The expression of layilin was upregulated in activated CD8+ T cells and Tregs and inhibited CD8+ T-cell function *in vitro* (11). In exhausted T cells and Tregs of liver cancer tissue, its TCRs are reused. The proportion of T cells containing the same TCR is higher in HCC tissues than in peripheral blood and normal tissues, suggesting that clone amplification occurs in exhausted T cells and Tregs in HCC tissues (12). CD4+ T cells have tumor-specific states, and multiple cytotoxic CD4+ T cells have been cloned and amplified. These CD4+ T cells kill autologous tumors in a major histocompatibility complex (MHC) class II-dependent manner and are inhibited by Tregs. In addition, the gene expression profile of cytotoxic CD4+ T cells in tumor tissues is associated with the clinical response of metastatic bladder cancer patients treated with anti- programmed death-ligand 1 therapy (13). The number of cytotoxic T cells and clonicity of the TCR are decreased in patients with squamous cell carcinoma after organ transplantation. Phenotypic function identification of T cells with these TCRs can promote the personalized treatment of skin squamous cell carcinoma with strong immunity (14). Tumor cells from nasopharyngeal carcinoma (NPC) patients show a high degree of intratumor and intertumor heterogeneity, and are characterized by T-cell clones and extended distribution of individual tumors, providing insights into the mechanisms by which immune cells clear tumors and improving NPC targeting and immunotherapy (15). CD8+ TILs and their TCR libraries may be the basis of antitumor immune responses in different hosts, which may have important implications for the development of personalized immunotherapies for cancer (16). T-cell large granular lymphocytic leukemia (T-LGLL) is a lymph proliferative disease characterized by the clonal expansion of terminal differentiation effector and memory cytotoxic T lymphocytes (CTLs). Abnormalities in cell survival and apoptotic gene programming and significant downregulation of CD8+T cell apoptotic genes are prominent features of T-LGLL cells (17). The studies on single-cell TCR/BCR sequencing technology applied to tumors are summarized in **Table 1**.

**Table 1:** Summary of the application of single-cell TCR/BCR sequencing technology in tumor research.

Disease	Technology	Significance	Reference
Acute myeloid leukemia	scRNA-Seq+ scTCR-Seq	Reveal tumor immune microenvironment	(6)
Breast Cancer	scRNA-Seq+ scTCR-Seq	Reveal tumor immune microenvironment	(7, 8)
Melanoma	scTCRSeq+scRNA-Seq	Provide clues for disease diagnosis and clinical management; monitor the ability of the blood to respond to anti-tumor CD8 ^+^ T cells	(9, 10)
Liver Cancer	scTCR-Seq	Reveal tumor immune microenvironment	(11, 12)
Bladder Cancer	scTCR-Seq	Predict clinical response to anti-PD-L1 therapy	(13)
Squamous cell carcinoma	scRNA-Seq+ scTCR-Seq	Facilitate personalized treatment of SCC	(14)
Nasopharyngeal carcinoma	scTCR-Seq	Improve targeted therapy and immunotherapy for NPC	(15)
Scale-cell carcinoma of head and neck	scRNA-Seq+scTCR-Seq	Guide personalized cancer immunotherapy	(16)
large granular lymphocyte leukemia T cells	scRNA-Seq+ scTCR-Seq	Reveal tumor immune microenvironmen	(17)

## Infectious diseases

A total of 41,718 CD3+ T cells have been identified in tuberculosis pleural effusion (TPE), and no difference in distribution has been observed in the CDR3 of CD4+ and CD8+ T cells. The hydrophobicity of CDR3 is changed in CD8+ T cells, and T cell receptor beta variable 4-1 (TRBV4-1) is preferentially expressed in TPE; the CD4+ T cell subpopulation may be important for protective immunity against tuberculosis (18). Previously, CD4+ T cells reactivated by cytomegalovirus (CMV) structural protein pp65 were isolated from human peripheral blood with significant heterogeneity and potential function. Tregs were the largest population of these reactivated cells. CD4+ CTL1 and CD4+ CTL2 cells reactivated by CMV were cloned and amplified; they share a large TCR library. This study provides clues regarding the function and interaction of CD4+ T-cell subsets during CMV infection (19). The dynamics and diversity of T-cell immune libraries in human immunodeficiency virus-negative pneumocystis pneumonia remain unclear. Single-cell sequencing in the lung tissues of mice infected with pneumocystis showed a decrease in TCR diversity of CD4+ T cells and an increase in CD8+ T cell diversity in mice infected with pneumocystis, providing clues to the mechanism of the host’s adaptive immune response to pneumocystis (20). Different T cell clones have been amplified in COVID-19 patients. Further analyses of the VJ gene(V-variable,J-joining) mix have revealed that among COVID-19 patients, 6 VJ pairs are significantly increased and 139 pairs are significantly reduced. These results contribute to further elucidating the mechanism of severe acute respiratory syndrome coronavirus 2 (SARS-COV-2)-induced immune responses (21). BCR diversity is significantly reduced in COVID-19 patients, and the CDR3 sequence of the BCR heavy chain is similar to that of healthy controls. Among all cloned BCRs, IgG isotypes have the most frequent class-switching recombination events and the highest rate of somatic super mutation, especially IgG3. This has important implications for elucidating the immune response mechanism of SARS-COV-2 infection (22). A characterization of peripheral blood T and B cell variation in COVID-19 patients shows that humoral immune response and T cell immune memory were positively correlated with disease severity (23). Asymptomatic COVID-19 patients showed an increase in CD56briCD16- natural killer (NK) cells and upregulation of interferon -γ in effector CD4+, CD8+ T cells and NK cells. They showed more robust TCR clone amplification, especially in effector CD4+ T cells, but lacked intense BCR clone amplification compared to moderate patients (24). The germinal center (GC) B-cell subsets and organ specificity of lymph nodes and spleen cells infected with influenza virus continue to differ during the response process, and there is significant clone overlap in GC-derived plasma cells. This provides important clues to understanding the mechanisms of immune responses against viruses (25). Cutaneous erythema migrants (EM) is the first sign of a tick-borne infection called Lyme disease. T cells and innate immune cells predominate in EM lesions and promote the response. B-cell cloning and amplification in the skin of EM patients and the expression of MHC class II genes in EM-associated B cells are upregulated. This provides a direction for revealing the mechanism of immune responses in borrelia infection (26). The application of single-cell TCR/BCR sequencing technology to infectious disease research is summarized in **Table 2**.

**Table 2:** Summary of the application of single-cell TCR/BCR sequencing technology in infectious diseases.

Disease	Technology	Significance	Reference
Tuberculosis	scTCR-seq+sc-RNA seq	Involve in protective immunity	(18)
Cytomegalovirus	scTCR-seq+sc-RNA seq	Elucidate the function and interaction of CD4 ^+^ T cells	(19)
Pneumocystis Pneumonia	scTCR-seq+sc-RNA seq	Reveal the mechanisms of host adaptive immune responses to pneumocystis	(20)
*COVID-19*	scTCR-seq+sc-RNA seq ;scBCR-Seq ; scTCR-Seq+ scBCR-Seq)	Involve in T-cell mediated viral clearance;Reveal the immune response mechanism of viral infection	(21–24)
*Borrelia* infection	scBCR-seq+sc-RNA seq	Reveal the immune response mechanism of viral infection	(25)
Lyme disease	scBCR-seq+sc-RNA seq	Reveal the immune response mechanism of borrelia Infection	(26)

## Autoimmune diseases

T helper type 1 (Th1) and Th17 cells activated in the peripheral blood of patients with primary Sjogren’s syndrome (pSS) express TCRβ variables (TRBV) 3-1/connector (J) 1-2 (CLFLSMSACVW) and TRBV20-1/J1-1 (SVGSTAIPP * T). TCRα variable 8-2/J5 (VVSDTVLETAGE) is expressed by the Th1 cells of pSS patients, and a CDR3α-specific motif (LSTD * E) was found in Th1/Th17 cells. This provides clues to elucidating the pathogenesis of pSS (27). CD8+ and CD4+ T cells are activated in the peripheral blood of patients with adenosine deaminase 2 deficiency, and T cells show significant cell-cell interaction with monocytes, which promote the upregulation of signal transducer and activator of transcription 1 (STAT1) expression in T cells (28). Immune cells, T cells, and B cells play an important role in the pathogenesis of systemic lupus erythematous (SLE). Sixteen immune cell types are present in the peripheral blood of SLE patients, and TCR and BCR types are increased, providing new approaches for the diagnosis and treatment of SLE (29). Orbital disease, the most serious manifestation of Graves’ hyperthyroidism (GH), is an autoimmune-mediated inflammatory disease with typically a low therapeutic effect. The CD4+ CTL population in the peripheral blood of patients with GH has clonal amplification. Their strong cytotoxic response to auto antigens and orbital localization are potentially associated with disease recurrence (30). Somatic mutations in clonally amplified CD8+ lymphocyte populations in patients with rheumatoid arthritis (RA) and unique TCRβ characteristics have been detected in patients with invasive destructive RA, who express high levels of tumor necrosis factor superfamily member 14 cytokines. The specific characterization of TCRβ in CD8+ T lymphocytes may help improve treatment regimens for patients with drug-resistant RA (31). In autoimmune hepatitis, the presence of autoantibodies against soluble liver antigen (SLA) is associated with reduced overall survival, but the associated auto reactive CD4 T cells have not been characterized. SLA-specific CD4T cells have been tracked in peripheral blood by single-cell sequencing. The results showed that: autoreactive SLA-specific CD4 T cells have memory PD-1+CXCR5-CCR6-CD27+ phenotype, and autoreactive TCR clones mainly exist in memory PD-1+CXCR5-CD4 T cells and induce B-cell differentiation through interleukin 21 (32). The application of single-cell TCR/BCR sequencing technology to autoimmune diseases is summarized in **Table 3**.

**Table 3:** Summary of studies on the application of single-cell TCR/BCR sequencing technology in autoimmune diseases.

Disease	Technology	Significance	Reference
Primary Sjögren's syndrome	scTCR-seq	Reveal pathogenesis	(27)
Adenosine deaminase 2	scRNA-seq+scTCR-seq	Reveal pathogenesis	(28)
Systemic lupus erythema	scTCR-seq+scBCR-seq	Provide a new approach for diagnosis and treatment of SLE	(29)
Graves' hyperthyroidism (GH)	scRNA-seq+scTCR-Seq	Provide potential therapeutic targets	(30)
Rheumatoid arthritis	scRNA-seq+scTCR-Seq	Develop improved protocols for resistance therapy in patients	(31)
Autoimmune hepatitis	scTCR-seq	Reveal pathogenesis	(32)

## Chronic inflammatory diseases

Although various pro- and anti-inflammatory T-cell subsets have been observed in human atherosclerotic plaques, the main question of T-cell immunity remains unanswered. T-cell transcriptome and TCR maps of three important tissues associated with atherosclerosis have been provided by single step-cell sequencing. This approach is expected to address major questions about atherosclerosis autoimmunity (33). Single-cell sequencing of pancreatic immune cells isolated from hereditary and idiopathic chronic pancreatitis (CP) patients undergoing total pancreatectomy has revealed reduced T-cell clonicity in hereditary CP, and C-C motif chemokine receptor 6 (CCR6) ligand (CCL20) expression is significantly upregulated in monocytes of hereditary CP. The CCR6-CCL20 signaling pathway may be used as a potential therapeutic target for human inherited CP (34). The detection of TCR+ macrophages, proliferative macrophages, and natural killer dendritic cells in peritoneal fluid of endometriosis by single-cell sequencing suggests that immune dysfunction occurs in the peritoneal fluid of endometriosis and provides a valuable tool for the future development of immunotherapy (35).

A new fibrotic subpopulation of CD8 T (CCL5 +, CCL4 +) and CD4 T (MT-CO1 +) cells infiltrate the fibrotic liver, characterized by the abnormal activation or inactivation and a marked decline in TCR clones, along with the reduced use of VJ and VDJ fragments. The pattern and dynamics of these individual immune cells in liver fibrosis contribute to elucidating the protective mechanism of TCR in the chronic liver injury response (36). The TCRα chain is significantly enriched in the blood of patients with Crohn’s disease (CD), particularly in CD8+ T cell populations, whereas the potential effects of Crohn’s associated invariant T-cell subpopulations on CD remains to be elucidated (37). A total of 1650 glutamic acid decarboxylase 65-kilodalton isoform (GAD65)-specific CD4(+) T cells were isolated and 1003 different TCRs were identified in the peripheral blood of 6 patients and 10 patients with type 1 diabetes mellitus who were positive for islet autoantibodies. The TRBV5.1 gene was most highly expressed in the GAD65 557I tetramer (+) cells, and these findings provide strong support for revealing the pathogenesis of type 1 diabetes (38). Mutations in the transcriptional regulator STAT3 lead to neonatal type 1 diabetes. Paired single-cell TCR and RNA sequencing has shown that STAT3 gain of function (GOF) drives significant proliferation of terminal depletion-resistant effector CD8+ cells. A single-cell assay for transposase accessible chromatin with high-throughput sequencing showed that these effector T cells are epigenetic and have different chromatin structure induced by STAT3-GOF, CD8+T cells react with known antigen islet-specific glucose-6-phosphatase catalytic subunit-related protein, STAT3 mutations contribute to type 1 diabetes through deficiency of CD8+ T cell tolerance (39). A large number of CD8+ T cells continued to progress from central memory to terminal effect in the peripheral blood of patients with Parkinson’s disease (PD), and cytotoxic CD4+ T cells (CD4 CTLS) were significantly amplified from Th1 cells, providing valuable insights and rich resources for understanding adaptive immune responses in PD patients (40). Another single-cell sequencing showed that the memory B cells of PD patients were significantly increased and the naive B cells were significantly decreased. The memory B cell population upregulated the expression of MHC II genes (HLA-DRB5, HLA-DQA2, and HLA-DPB1) and transcription factor activator protein 1, and the antigen presentation ability of B cells of PD patients was enhanced. The results provide new insights into humoral immune responses in the pathogenesis of PD (41). The studies on the application of single-cell TCR/BCR sequencing technology to chronic inflammatory diseases are summarized in **Table 4**.

**Table 4:** Summary of studies on the application of single-cell TCR/BCR sequencing technology in chronic diseases.

Disease	Technology	Significance	Reference
Atherosclerosis	scRNA-seq+scTCR-seq	Reveal the mechanism of immune response	(33)
Chronic pancreatitis	scTCR-seq	Reveal the mechanism of immune response	(34)
Endometriosis	scRNA-seq+scTCR-seq	Reveal the mechanism of immune response	(35)
Hepatic fibrosis	scTCR-seq	Reveal the mechanism of immune response	(36)
Crohn's disease (CD)	scRNA-seq+ scTCR-seq	Reveal the mechanism of immune response	(37)
Type 1 diabetes	scRNA-seq+ scTCR-seq	Reveal the mechanism of immune response	(38, 39)
Parkinson's disease	scRNA-seq+ scBCR-seq	Reveal the mechanism of immune response	(40, 41)

## Discussion

Immune library sequencing (single-cell TCR/BCR sequencing) can solve the following problems: identify the sequence composition and diversity of the immune repertoire; explore gene expression and discover new biomarkers; and analyze clonotype composition within/between samples (e.g., proliferative cloning, clonotype overlap between cell types, clonotype diversity) and clonotype composition between samples (e.g., clonotype overlap between different samples of the same organism). Single-cell immunosequencing can simultaneously detect thousands of cells in a single experiment, and the 5’-end mRNA expression profile as well as TCR and BCR information can be obtained simultaneously in a sample. Combined with gene expression profiles and V(D)J data, factors influencing immune responses in complex tissue samples are analyzed. However, single-cell TCR/BCR sequencing technology also has the following shortcomings. 1) Stringent sample requirements: the initial number of cells in a single sample should be 10^5^ to 10^6^, and the number of living cells should be more than 80%, and it is recommended to be more than 90%. 2) At present, the proportion of T cells or B cells in tissue samples is not very large. If they are not separated from tissues, the TCR or BCR data will be less abundant.

The development of single-cell sequencing technology has promoted the development and maturity of immunobank sequencing technology. The increasing amount of immunobank data requires efficient analysis technology to realize the rapid and accurate analysis of high-throughput data so that cell heterogeneity in complex immune systems can be explored in a more in-depth and detailed manner. In addition, immunomics research can promote the development of cancer immunotherapy. Exploring early cancer screening from immunomics data is of great significance for cancer treatment, and the richness of the immune library between groups and individuals can directly reflect the body’s immune system. Generally, the more subtypes of TCR/BCR, the stronger the organism’s ability to identify pathogens, and the less susceptible it is to diseases. However, beyond a certain limit, it is also prone to cause autoimmune diseases. In addition, the body’s immune library is not invariable and its diversity is constantly changing with age, environment, drug use, diseases, and other factors. Therefore, the application of immunobank sequencing technology in clinical diagnosis and treatment is helpful for immune monitoring between groups or individuals, exploring the relationship between related diseases (e.g., tumor, disease infection, treatment, and autoimmune diseases) and immune responses, monitoring the effect of immunotherapy, and studying the molecular mechanisms of disease onset and progression.

## Author contributions

JHH and JS wrote the manuscript. TP, ZHG and LYL analyzed the data. WFL, ZPH and FMX revised the manuscript. JHL,YGL and HWC provided the funding. All authors read and approved the final manuscript.

## Funding

This work was supported by Medical Science and Technology Research Project of Guangdong Province (No. A2022524; A2020304), Science and Technology Program of Guangzhou (No. 202201010840; 202201010810; 202102080532; 202002030032; 202002020023), Health Commission Program of Guangzhou (20212A010025; 20201A010085), Science and Technology Project of Panyu, Guangzhou (2021-Z04-053; 2020-Z04-026; 2019-Z04-02), Scientific Research project of Guangzhou Panyu Central Hospital (No. 2022Y002; 2021Y004; 2021Y002).

## Conflict of interest

The authors declare that the research was conducted in the absence of any commercial or financial relationships that could be construed as a potential conflict of interest.

## Publisher’s note

All claims expressed in this article are solely those of the authors and do not necessarily represent those of their affiliated organizations, or those of the publisher, the editors and the reviewers. Any product that may be evaluated in this article, or claim that may be made by its manufacturer, is not guaranteed or endorsed by the publisher.

## Glossary

**Table:**

NK-AML	normal karyotype acute myelogenous leukemia
snRNA-seq	single-cell transcriptome sequencing
scRNA-seq	single-cell RNA-sequencing
scTCR-seq	single-cell TCR/BCR sequencing
scBCR-seq	single-cell BCR sequencing
snRNA-seq	single-nucleus RNA sequencing
IR	immune repertoire
IR-SEQ	Immune repertoire sequencing
BCR	B-cell receptor
TCR	T-cell receptor
CDR	complementary determining region
MAIT	mucosal-associated invariant T cell
TIL	tumor-infiltrating lymphocyte
ICI	immune checkpoint inhibitor;
irAE	immune-related adverse event
HNSCC	head and neck squamous cell carcinoma
NPC	nasopharyngeal carcinoma
TPE	tuberculous pleural effusion
HIV	human immunodeficiency virus
PCP	pneumocystis pneumonia
EM	erythema migrans
pSS	primary Sjögren’s syndrome
RA	rheumatoid arthritis
SP-RA	seropositive rheumatoid arthritis
SN-RA	seronegative rheumatoid arthritis
WES	whole exome sequencing
AIH	autoimmune hepatitis
SLA	anti-soluble liver antigen
CP	chronic pancreatitis
CD	Crohn’s disease
T1D	type 1 diabetes
GOF	gain-offunction;
PD	Parkinson’s disease
HLA-DRB5	human leukocyte antigen-DRB5
HLA-DQA2	human leukocyte antigen-DQA2
AP-1	HLA-DPB1 transcription factor activating protein
cDNA	complementary Deoxyribonucleic acid
